# A new method for measuring the psychoacoustical properties of tinnitus

**DOI:** 10.1186/1746-1596-8-209

**Published:** 2013-12-19

**Authors:** Bozena Kostek, Tomasz Poremski

**Affiliations:** 1 Audio Acoustics Laboratory, Faculty of Electronics, Telecommunications and Informatics, Gdansk University of Technology, Gdansk, Poland; 2 Training and Development Department, GEERS (GEERS Hearing Acoustics), Narutowicza 130, 90-146 Łódź, Poland

**Keywords:** Tinnitus testing, Multimedia-based sound synthesizer, Audiometer, Psychoacoustics measurements of tinnitus characteristics

## Abstract

**Background:**

This study investigates the usefulness and effectiveness of a new way of tinnitus screening and diagnosing. The authors believe that in order to arrive at relevant diagnostic information, select the tinnitus treatment and quantitatively substantiate its effects, the measurement of the Tinnitus psychoacoustic parameters should be made an inherent part of the Tinnitus therapy.

**Methods:**

For this purpose the multimedia-based sound synthesizer has been proposed for testing tinnitus and the results obtained this way are compared with the outcome of the audiometer-based Wilcoxon test. The method has been verified with 14 patients suffering from tinnitus.

**Results:**

The experiments reveal capabilities, limitations, advantages and disadvantages of both methods. The synthesizer enables the patient to estimate his/her tinnitus more than twice as fast as the audiometer and makes the information on the tinnitus character perception more accurate. The analysis of the Wilcoxon test results shows that there are statistically important differences between the two tests.

**Conclusions:**

Patients using the synthesizer operate the software application themselves and thus get more involved in testing. Moreover, they do not concentrate on describing verbally their tinnitus, which could be difficult for some of them. As a result, the test outcome is closer to the perceived tinnitus. However, the more complex the description of the perceived tinnitus, the harder it is to determine the sound parameters of the patient’s perception. It also takes more time regardless of the method.

**Virtual slides:**

The virtual slide(s) for this article can be found here: http://www.diagnosticpathology.diagnomx.eu/vs/1954066324109436

## Abstract (de)

**Grundinformationen**

Das Ziel der vorliegenden Arbeit ist es, die Nützlichkeit und die Effektivität einer neuen Art von Screening und Diagnostik von Ohrgeräuschen (Tinnitus) zu untersuchen. Die Messung der psychoakustischen Parameter von wahrgenommenen Ohrgeräuschen soll ein Therapieteil sein, damit entsprechende Informationen für die Diagnostik, für die Wahl der entsprechenden Therapie sowie für die quantitative Bestimmung deren Ergebnisse gewonnen werden können.

Um Tinnitus-Parameter zu bestimmen, wurde ein multimedialer Tonsynthesizer verwendet. Die gewonnenen Ergebnisse wurden mittels dem Wilcoxon-Test mit denen verglichen, die mittels einem klinischen Audiometer erhalten wurden. Diese Methode wurde an 14 Tinnitus-Patienten erprobt.

Die durchgeführten Untersuchungen zeigen Möglichkeiten, Einschränkungen, Vor-und Nachteile der beiden Methoden. Der Synthesizer ermöglicht dem Patienten, die Tinnitus-Parameter über zweimal schneller einzuschätzen, auch die Informationen über die Wahrnehmung von Ohrgeräuschen sind genauer. Die Ergebnisse der Analyse, die sich auf den Wilcoxon-Test stützt, zeigen, dass es einen statistisch wichtigen Unterschied zwischen der Länge der Untersuchungsdauer gibt, die mit dem Audiometer und dem Synthesizer durchgeführt werden.

**Fazit**

Die eigene Bedienung des Synthesizers durch den Patienten bewirkt, dass er sich in die Untersuchung mit einbezogen und engagiert fühlt. Darüber hinaus konzentriert er sich nicht an die verbale Beschreibung des wahrgenommenen Ohrgeräusches, was für einige Patienten schwierig ist. Deshalb ist das Ergebnis des bezeichneten Tinnitus an das wahrgenommene Klingeln der Ohren am nächsten. Je komplexer die Ohrgeräusche jedoch sind, desto größere Schwierigkeiten haben die Patienten, die Eigenschaften zu beschreiben, was dazu führt, dass es schwieriger ist, die Parameter der wahrgenommenen Ohrgeräusche zu bekommen. In solchen Fällen nimmt die Untersuchung mehr Zeit in Anspruch und das ist methodenunabhängig.

## Background–psychoacoustic measurements of tinnitus characteristics

The first step of the tinnitus therapy should include the measurement of the psychoacoustic parameters of tinnitus to get relevant diagnostic information, select a treatment and quantitatively substantiate its effects. However, the clinical relevance of these measurements depends on the form of the applied treatment or therapy. As indicated by Henry & Meikle and Schechter & Henry [1,2], in the case of the tinnitus therapy based on masking sounds, the key is to measure and document the impact of the masking stimuli on the perception of tinnitus. For this purpose, it is helpful to measure the minimum masking level (MML). However, other parameters, such as loudness, pitch matching and residual inhibition may also be useful in the classification of subjective tinnitus [3]. Jastreboff [4] believes that also the measurement of the tinnitus parameters is generally important in terms of individual consultations with patients undergoing TRT therapy (Tinnitus Retraining Therapy). Tyler et al. [5] point out that the measurement of the tinnitus parameters is justified if it is used in a treatment plan. Jastreboff and Hazell [6] state that these parameters are not only associated with the subjective intensity tinnitus is felt or with its severity. They disclose the changes connected with reduced tinnitus perception, that could be helpful during consultation with patients. Psychoacoustic measurements are also valuable while assessing and verifying patients’ subjective reports on the state of their tinnitus.

The need for the psychoacoustic evaluation of tinnitus was first mentioned in 1903 [7]. But only when an appropriate electroacoustic equipment was invented, it was possible to match pure tones with the loudness and pitch of tinnitus [8-10]. Another approach to assessing tinnitus was proposed by Fowler. First, he described a method of measuring loudness recruitment that is based on equalizing the sensation of loudness between both ears in order to get the same impression of sound intensity called ABLB (Alternate Binaural Loudness Balance) [11,12]. Next, he used the ABLB approach as a test for equalizing the sensation of loudness of tinnitus in one ear with the loudness of a tone fed to the opposite ear [13]. The loudness of the comparison tone, expressed in dB SL (Sensation Level), is an indicator of the tinnitus volume experienced by a patient. Fowler [14] believed that it was crucial to achieve a tone corresponding to the tinnitus in the opposite ear. It should be noted here that in practice this is only possible when tinnitus is heard by the patient on one side only. Another observation made by Fowler [15,16] was that even patients who report their tinnitus as very loud usually define the loudness of the tone fed to them at a level of 5-10 dB SL only. This observation is also confirmed in the study conducted by the authors of this paper. Fowler’s basic method, or combinations of it, have been used in a number of subsequent studies and publications [17-22].

Attempts to determine the criteria for assessing tinnitus were made by the Ciba Foundation in London [23] and the National Academy of Sciences [24]. Both served as the basis for creating recommendations for a set of clinical trials with a view to describing tinnitus, which included: loudness matching, pitch matching, tinnitus maskability and residual inhibition. The term ‘maskability’ refers to the degree to which tinnitus may be covered up or “masked” by other external signals. Details and procedures for implementing the clinical trials were developed by Vernon and Meikle [25]. These involve three separate, consecutive procedures, which consist of threshold testing and determining the loudness and pitch of tinnitus. As stated by Henry et al. [26], these procedures were not adopted as clinical standards for tinnitus assessment at that time, mainly due to the requirement for specialized diagnostic equipment.

In his studies, Henry at al. [27,28] describes a method for determining the loudness and pitch of tinnitus that may be carried out with a clinical audiometer. The procedure is as follows: once the audiogram has been made with the use of pure tones, the examiner goes on to obtain the best match between the comparison pure tones and the pitch and loudness of the tinnitus. At the beginning, the tones used for loudness matching should be fed at 10-20 dB SL within the range of normal hearing threshold, and at 5-10 dB SL within the range with hypacusia (diminished acuteness of the sense of hearing). The loudness of the tone fed in this way will vary depending on the degree of hearing loss and its perception in relation to the loudness of the tinnitus. Another method used to assess the most prominent pitch of the tinnitus introduced by Vernon and Fenwick [29] is based on a two-alternative forced-choice (2AFC) procedure. According to its creators, it is more accurate than the previous methods. However, this method has frequently been criticized in the literature. The procedure involves presentation of two tones of different frequencies. The patient decides which of the presented tones is closer to the frequency of tinnitus.

The first estimation of tinnitus pitch should be made for the octave frequencies, and the test should start at a frequency of 1000 Hz. After setting the first tone at a frequency of 1000 Hz, the person conducting the examination should set the tone close to the tinnitus loudness level as perceived by the patient. Then, the loudness of the tinnitus should be determined. Then, the examiner attempts to determine whether tinnitus is higher or lower than the tone fed. For most patients, the 1000 Hz tone will be lower than the perceived tinnitus. The procedure is performed in this way as long as the pitch of the tinnitus is narrowed to one octave. Then, the same method is applied to determine the tinnitus loudness and pitch for inter-octave frequencies. The pitch of the tinnitus is thus determined at the most with an accuracy of a half-octave. Obviously it depends on the capacity of the audiometer used.

Patients often have difficulty in determining the pitch of the tinnitus they hear in relation to the frequency of the tone fed [30]. Their task is to specify a tone that is as close as it is possible to the tinnitus heard. In this case, tones of one octave below and one octave above are given to make this comparison simpler. The final determination of the pitch of the tinnitus should be made when feeding a tone with the loudness as close as it is possible to the perceived tinnitus. This is why, in accordance with the procedure described by Vernon and Meikle [25], one should use the lowest available level resolution of the audiometer.

The studies conducted by the Authors of this paper used the method described by Vernon and Meikle [25], however, a modified procedure has been used in which the test sequence was reversed. This happens to be in accordance with the instructions given by Schwartz [31]. To be more exact, first it was attempted to find a tone the frequency of which was as close as it is possible to the pitch of the perceived tinnitus, and only then did they determine its loudness. It was decided to follow this sequence since each loudness equalization between the tone and tinnitus significantly prolonged the process of obtaining the relevant tinnitus parameters.

As described by Henry [27], the next step after obtaining the pitch and loudness of the tinnitus is to determine its nature, i.e. whether it sounds more like a tone or more like noise. Although many patients are able to adjust their tinnitus to the tone fed by an audiometer, it is not usually identical to their own tinnitus. This is because audiometers are only able to generate a limited range of sounds such as simple tones, and narrowband and broadband noise. If the tinnitus reported by the patient more closely resembles noise, the audiometer should feed a narrowband noise with a centre frequency equal to pitch of the tinnitus obtained earlier with pure tones to the patient. If the patient reports that the tone was more similar to their tinnitus than the noise, the result obtained with the tone is the final result and there is no need for further tests with noise for other octave frequencies. However, if the noise is closer to the tinnitus, a check should be performed to see what kind of noise has the best match. Broadband noise may be fed (speech noise or white noise) and alternated with narrowband noise for this purpose. In this way, the patient has at least three stimulation options to choose from in arriving at the best tinnitus description: pure tone, narrowband noise and broadband noise. The loudness is re-matched for the type of noise selected by the patient in the smallest possible increments (e.g. 1 dB) to determine the best tinnitus match.

Due to the limitations imposed by the capabilities of audiometers, defining the tinnitus parameters is quite time-consuming and does not always yield satisfactory results in the form of a match with the tinnitus that is actually perceived by the patient. Another hindrance is the need for the patient to give answers that require them to subjectively balance their own noise with the sound generated by the clinical audiometer. Moreover, this is the test administrator that makes changes in the parameters of the presented signals based on the patient’s feedback. Therefore, it is necessary to establish good contact with the patient.

Henry suggested that the noise presentation should begin with the narrowest band for the frequency corresponding to the tinnitus pitch obtained with pure tone if there exists a device allowing the continuous adjustment of the fed noise bandwidth, [27,28]. If the similarity of the noise is better than that of the tone, its bandwidth should be gradually extended until the best match is obtained.

Another psychoacoustic parameter which may be useful in evaluating tinnitus treatment is the measurement of the minimum masking level (MML). This is the minimum level of broadband noise at which the patient’s individual tinnitus is inaudible. MML is a commonly-used measuring method applied by many clinics dealing with tinnitus and regarded as correlating with the effectiveness of treatment [32]. This means that if patients indicate improvements (reduction of tinnitus perception), a decrease in MML follows. It is important that the MML is not seen as a general indicator of the effectiveness of the therapy and it is used as an indicator only when the treatment incorporates tinnitus masking sounds. In accordance with the observations [33,34], it is with this type of therapy that the level difference between MML and the loudness of tinnitus indicates the effectiveness of the therapy. When the MML is smaller than the loudness of the tinnitus, masking sound therapy will probably be beneficial. However, when the MML is higher than the loudness of the tinnitus, this type of therapy is less likely to be advantageous.

Henry [27] reports that MML clinical trials can be performed with the use of either monaural or binaural stimulation. However, the patient often finds monaural stimulation cumbersome. That is why Henry recommends binaural stimulation for routine tests. For most patients tinnitus masking occurs up to 10 dB SL [28]. Broadband noise and the patient’s hearing thresholds are used to determine the MML. First, the noise is set to the hearing threshold level for one ear and then to the hearing threshold level for the other ear. After determining the noise level for both ears, the noise is increased in 1 dB increments until the patient reports that the tinnitus is inaudible. If, during the test, the patient reports that the tinnitus has become inaudible in one ear only, the noise is increased contralaterally until the tinnitus is completely inaudible.

Psychoacoustic evaluation of tinnitus can be extended, especially in case of auditory hypersensitivity. To determine the degree of auditory hypersensitivity the LDL (Loudness Discomfort Level) or UCL (Uncomfortable Level) should be measured using live speech or pure tones. If an individual experiences simultaneously different types of tinnitus, one should measure and concentrate on the noise characteristics of the most annoying MTT (Most Troublesome Tinnitus) [35].

In therapies which use sound stimulation (e.g., noise stimulation) a parameter known as residual inhibition can be measured. This defines a temporary reduction or total elimination of tinnitus perception as a result of sound stimulation [36-38]. This phenomenon was formally described by Feldmann [39] in 1971, although it had been recognized in earlier studies [7,9].

Residual inhibition evaluation may be used to assess the results of the therapy. Henry et al. [28] describe the use of this method as a continuation of the MML test. In this case, stimulation employs the same type of broadband noise that was used to determine MML + 10 dB. The patient is told that after a 1-minute exposure to the noise he or she is to specify whether his or her perception of the tinnitus changes in any way. If there has been a reduction in the perception, the result is recorded as a percentage. It reflects the extent to which the perception of tinnitus was successfully reduced in relation to its normal perception. Residual inhibition measurement was also used in other research we conducted previously. It involved the synthesizer design, in particular in the study of the influence of ultrasonic noise on the perception of tinnitus [40].

The first attempts to standardize the methods of measuring psychoacoustic parameters were made at the beginning of the 1980s. However, they have not been fully harmonized yet. As a result, research centers dealing with tinnitus assessment have developed their own individual procedures for determining tinnitus. Because of lack of standardization in this area, it is often impossible to compare results between different centers and clinics.

It is easy to notice that providing a masking noise to the tinnitus sufferers stops the process of spontaneous noise generation caused by the threshold characteristic. The efficiency of such elimination techniques developed for ear may be a good justification for the interpretation that defines ear noise as a direct consequence of weak audio signals quantization in threshold circuits. Treating the hearing system as an acoustic transmission chain, one can employ the general theory of spontaneous noise generation to search for new interpretations on ear noises origin. The pathogenesis of tinnitus may then be modeled using analogy to the digital transmission channel. When the amplitude of the quantified signal is close to that of the quantization threshold, the spectrum of the processed signal shows significant harmonic interference. The quantization error may have a value as high as the quantization threshold itself. As a result of interpreting the hearing loss as the increase in the quantization threshold, this threshold may appear on a level that is very high in comparison to the full scale of hearing dynamics. Such quantization causes severe frequency distortions of the signal. In digital transmission channels in such cases a dithering technique is applied, which consists in low level ultrasonic noise. Such an approach to Tinnitus induces new more effective methods of diagnosing and treatment, which can then be called an “ear dithering” [41]. The above mentioned issues are thoroughly discussed in the monograph by Czyzewski A., Kostek B., Skarzynski H., “*Application of computer technology to audiology, phoniatry and speech therapy*” published by Academic Press, Exit, Warsaw [41], and also in Czyzewski’s paper [42]. The conducted analysis shows how the interpretation of noise origin in quantizing circuits and its elimination through the means of additional masking noise (dither) may be used to explain the phenomena related to ear noises. But, not until recently the Authors developed means to justify this novel theory on tinnitus mechanism [40].

While diagnosing tinnitus, a series of tests is carried out to determine the source, location and the causes of the tinnitus. One of the most important parts is the interview that requires the patients to identify and describe their tinnitus listening experience. The description of the tinnitus characteristics may include information on its type and classification, which can then be used to plan further diagnosis or treatment. For the follow-up inquiry, the patient is provided with sounds closely resembling his or her tinnitus experience. For the most part, the sound is generated by an audiometer or sample sounds are played by other media.

Since the possibilities for generating sounds in this way are limited, such a test takes time and requires skill in operating the diagnostic equipment. What is more, the sounds presented usually differ significantly from the perceived tinnitus, which has also been proved in studies conducted by other authors [35]. This is an important part of the test as some of the types of tinnitus may immediately suggest their etiology and the way to proceed in further treatment. It is assumed [35] that pulsatile tinnitus is often of vascular origin. It is also presumed that it is often monaural, its incidence is consistent with cardiac function and its severity can be affected by physical effort.

Tinnitus of mechanical origin is largely non-pulsatile. It produces noise that is perceived as a regular or sudden cracking, or as a ‘clock ticking’, when it is connected with myocloni, the obstruction of auditory tubes or muscle contractions. Degenerative changes of the temporomandibular joint may cause grinding and cracking sounds while chewing or opening the mouth. This type of tinnitus, referred to as an objective tinnitus, is treated by eliminating the specific nosological unit which triggers it. Treatment for both vascular tinnitus and mechanical tinnitus usually involves surgery. Other forms of tinnitus, characterized as subjective tinnitus, are not fit for causal treatment and are extremely varied in their nature. Patients most often describe these forms as squeal, wheeze, whistle, hum, the sound of running water or the murmur of the sea.

## Methods

The aim of this research study is to determine the usefulness and effectiveness of using the synthesizer in the diagnosis and screening of tinnitus. For this purpose a computer-based tool which has been developed at the Multimedia Systems Department, Gdansk University of Technology can be used in a relatively easy way to make an attempt to sound synthesis, which corresponds to perceived Tinnitus and helps to determine the frequency and characteristics of Tinnitus [43,44].

The idea of the tool for the synthesis of Tinnitus is based on a relatively simple mechanism of the sound generator, which has the following features:

•simple tone generation at any frequency and amplitude,

•white noise generating and filtering,

•AM modulation of any tone,

•any digital sound filtering

As mentioned above, in the devised generator, there are two sound sources: pure tone and noise. However, it is also possible to generate multi-tones. Since the sample rate of 44.1 kHz is used, then the bandwidth supports sound frequencies from 1 Hz to 22 kHz. The buffer length is 3 seconds. A straight-forward way of getting noise sample is to employ a random number generator, however instead of utilizing pseudorandom noise, a white noise sample was generated in the Adobe Audition application. To get a narrow-band filtered noise the FIR (Finite Impulse Response) filter was employed. The algorithm works as a multi-band equalizer. The whole frequency band is divided into of 62 1/8 octave sub-bands.

The difficulty of implementing such a synthesis is related to developing the user interface that would allow the synthesis without requiring the user’s knowledge and skills in the domain of audio processing. The interface should attract the attention of the user and should have a intuitive operation [43]. Such assumptions have been used while designing the user interface for Tinnitus diagnosis.

**Figure 1:**
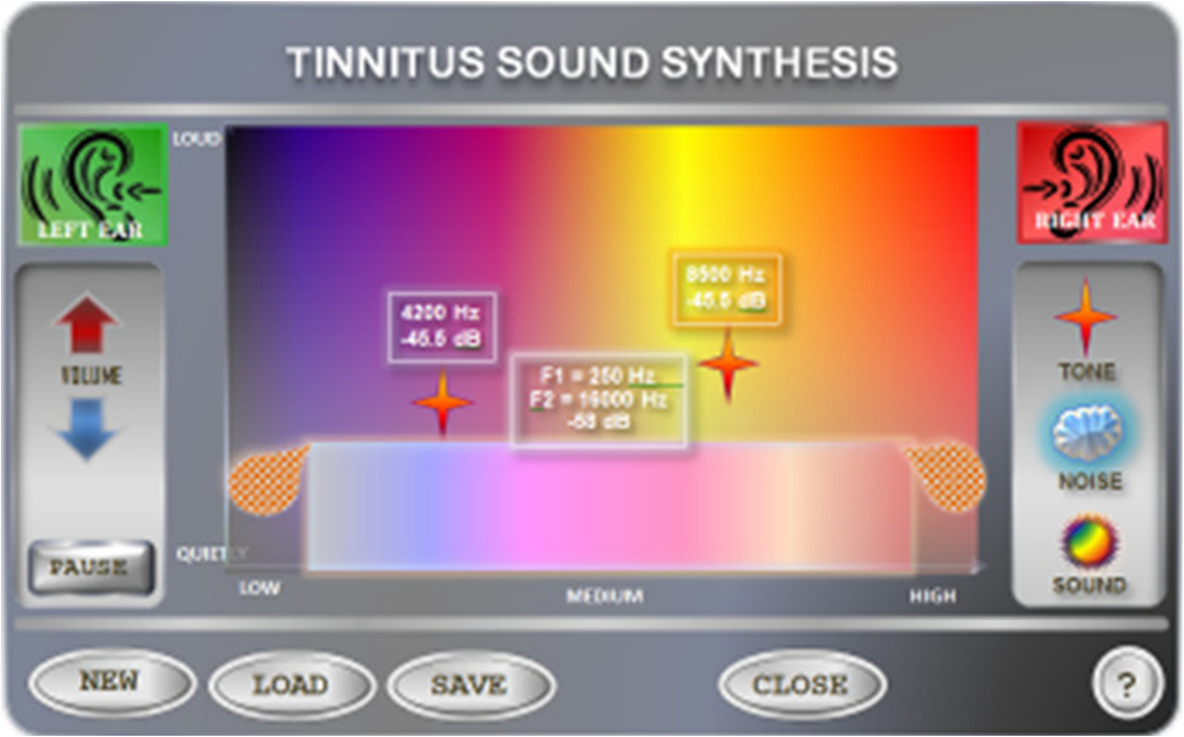
The user interface for Tinnitus sound synthesis.

The central element of Tinnitus synthesizer interface is a rectangular color space with axes marked at the bottom and on left side (Figure 1). The lower axis represents frequency of sound, while the vertical axis represents amplitude of sound. Amplitude as a function of frequency, i.e. amplitude spectrum is displayed in the window. There are three icons located on the right side of the panel. Each icon represents a different type of sound: a simple tone, white noise, sound. The user can select an icon and drag it to the above-described area. Moving icons horizontally will change sound frequency, while the vertical movement changes its amplitude. The designated area is assigned to each frequency band of different colors: cool colors to low frequencies, and warmer colors to higher frequencies. The color intensity represents the amplitude of sound (intensity)–the higher the sound level, the greater the intensity of color (more saturated). In case of simple tones the user modifies their frequency, whereas in the case of noise the user can adjust the frequency band to which the sound is limited in the frequency domain.

Moreover, people suffering from Tinnitus can often specify whether the sound has constant characteristic or is periodically changed. Very often, such a change at the time of the perceived sound is described by patients as a pulsing sound. This effect can be achieved by amplitude modulation (AM). Therefore, when the user double clicks a sound object, the window appears, where the speed, “throbbing” and its intensity (depth) can be set. To facilitate the scaling, the slider was described by relevant labels such as slow, fast or weakly, strongly.

The user during the Tinnitus synthesis may utilize any number of tones and noises. The synthesized sound can be stored to disk as a simple WAVE file format, as well as a project file that can be re-loaded for further modification.

It is recommended to monitor the synthesis result by listening to it, especially in cases of subjective Tinnitus. If Tinnitus occurs subjectively inside the head or in both ears, then the process of synthesis could be much more difficult. In the latter case, however, the synthesizer enables generating sound in one or both ears. The same sound is then heard in both ears. When tinnitus occurs in both ears but is of different character, measurements should be performed separately for each ear. Most of the patients taking part in measurements have tinnitus in both ears. This implies ipsilateral auditory stimulation. To compare all results on the same basis, such ipsilateral procedure was applied to all patients no matter whether tinnitus occurred in one or in both of their ears.

The tests of the effectiveness of synthesizer application involved 15 people suffering from tinnitus of varying etiology. In further stages of this study, these patients were also involved in evaluating the effectiveness of ultrasound linearization in the treatment of tinnitus.

Prior to the study, the participants are interviewed and are given audiometric tests, such as: air and bone tone audiometry, otoacoustic emissions (DPOAEs) and tympanometry with designation of stapes responses. Subjects were from various cities, that’s why to ensure the same test conditions a special audiometric cabin was used cabins are the standard equipment available for conventional audiometric test rooms. In two cases (nos. 13 and 14 from Table 1), the examination was performed in an anechoic chamber of the Laboratory of Audio Acoustics of the Gdansk University of Technology. Thus in all cases the conditions were similar and sound proof. The tests were performed in two stages. In the first stage the tinnitus level was assigned with the use of the Interacoustics AD 229E audiometer. In the second stage, the synthesizer was employed. The patients were divided into two groups for the examination. One of the groups used first the audiometer, and then the synthesizer. The other went through the tests in a reverse order. This was to avoid a systematic error that could occur for the same sequence of tests.

**Table 1:** Comparison of the results of determining tinnitus acoustic parameters using the audiometer and the synthesizer

**Interview**				**Stage I**			**Stage II**		
Participant no.	Gender	Age	Type and location of tinnitus (info from interview)	Tinnitus audiometric parameters [frequency|dB SPL]	Evaluation of the resemblance of the generated sound to the participant’s tinnitus in scale of [0…10]	Test duration [min]	Tinnitus parameters obtained with the synthesizer [frequency|dB SPL]	Evaluation of the resemblance of the generated sound to the participant’s tinnitus in scale of [0…10]	Test duration [min]
1	M	51	Squeal, in the head	RE: 6 kHz|65	**7**	**4**	RE: 2724 Hz|35.5	**9**	**2**
				LE: 4 kHz|59.5			LE: 2655 Hz|32.4		
2	M	67	High whistle, in LE and RE	RE: 4 kHz|34.5	**6**	**6**	RE: 6172 Hz|79	**9**	**2**
				LE: 4 kHz|34.5			LE: 6251 Hz|76.7		
3	M	77	Hum of bees, in the head	RE: 3 kHz|70	**3**	**4**	RE: 1929 Hz|56.7	**8**	**2**
				LE: 3 kHz|75			LE: 1954 Hz|57.3		
4	F	79	Low-frequency hum and squeal, in LE	LE: 2 kHz|49	**2**	**5**	LE: 82 Hz|88.4	**9**	**4**
							361 Hz|65.5		
							1281 Hz|39		
5	M	36	Squeal, in RE	RE: 4 kHz|79.5	**3**	**8**	RE: 5572 Hz|72	**3**	**6**
6	F	61	Murmur, squeal, in RE and LE	RE: 8 kHz|68	**6**	**10**	LE: Noise(8.9-10.4)	**9**	**8**
				LE: 8 kHz|63			kHz ; F_avg_ = 9682 Hz|44.5		
7	F	61	High metallic sound	RE: 8 kHz|33	**7**	**6**	RE: 7103 Hz|37.2	**9**	**2**
				LE: 8 kHz|43			LE: 7103 Hz|44.9		
8	F	65	Constant noise in head	RE: undefined	**9**	**6**	RE: undefined	**10**	**1**
				LE: 1 kHz NB|38			LE: Noise(0.35-2.1)		
							kHz; F_avg_ = 1229 Hz|31.6		
9	M	42	Squeal	RE: 4 kHz|59.5	**8**	**5**	RE: 3216 Hz|61.5	**8**	**2**
				LE: 3 kHz|60			LE: 3216 Hz|61.5		
10	F	61	Constant noise in RE	RE: 2 kHz NB|86	**8**	**3**	RE: Noise(0.9-2.4)	**5**	**4**
							kHz; F_avg_:1655 Hz|52.2		
11	F	37	Squeal or hiss in LE	LE: 3 kHz|35	**5**	**3**	LE: 2398 Hz|32.7	**5**	**2**
12	F	37	Squeal or hiss in LE	LE: 6 kHz NB|25	**7**	**5**	LE: Noise(6.2-6.6)	**8**	**3**
							kHz; F_avg_ = 6424 Hz|22.1		
13	M	24	High squeal or noise of working TV in head	RE: 6 kHz|20	**7**	**9**	RE: 6093 Hz|28	**8**	**4**
				LE: 6 kHz|30			LE: 6093 Hz|38.7		
14	M	24	High squeal or noise of working TV in head	RE: 6 kHz NB|20	**1**	**2**	RE: Noise(3.6-6.1)	**9**	**6**
							kHz; F_avg_ = 4874 Hz|14.3		
				LE: 6 kHz NB|35			LE: Noise(3.6-6.1)		
							kHz; F_avg_ = 4874 Hz|28.2		

Denotations: RE–right ear, LE–left ear, F_avg_–frequency average of noise, NB–audiometric narrow band noise, F–female, M–male.

Then, using a description of the tinnitus listening experience, the actual part of the test follows. As mentioned before, it has two stages:

I. Presenting simple tones available in the audiometer or narrowband noises with different frequencies. The audiometer is operated by a qualified person who, based on the subject’s responses, presents sample sounds which resemble the perceived tinnitus as closely as it is possible. At this stage, in addition to cooperating with the person conducting the test, the participant is asked to evaluate subjectively the resemblance of the generated sound to their own tinnitus on a scale of 0 to 10, or based on a percentage scale, that is, from 0% to 100%. The duration of the test is also one of the parameters to be assessed. This is measured from the start until the subject states which of the presented sounds is closest to their own tinnitus. Simple tones with the following frequencies can be used: 125, 250, 500, 750, 1000, 1500, 2000, 3000, 4000, 6000 and 8000 Hz, and the corresponding narrowband noises used commonly for masking in audiometric testing.

II. The participants determine the tinnitus parameters themselves using the synthesizer touch interface. The participants can choose from simple tones in the entire audible range (16 Hz-20 kHz) and white noise, which may be limited by band. These stimuli can be combined or used separately. The task of the participant is to set the sound that is close to their own tinnitus in terms of frequency and intensity. Just as in the first stage, the participants also have to determine the subjective resemblance of the generated noise to their tinnitus. The test duration is also measured.

Evaluation of the effectiveness of the synthesizer in determining tinnitus acoustic parameters involves comparison between the results obtained with the audiometer and the synthesizer. The comparison measures are the duration of the various test stages and the subjective evaluation of tinnitus patterns obtained with the two methods.

## Results–statistical analysis

The results of the Tinnitus estimation obtained based on synthesizer and audiometer are shown in Table 1. Table 1 includes information on the subjects’ gender and age. The group of patients consisted of seven women and seven men, the average age was 51.6 years for women and 46 for men (SD ± 5). In order to help in the evaluation of the research, all the results in the form of acoustic parameters for tinnitus have been unified and converted to decibels SPL [dB SPL]. The tests conducted by the authors showed that most patients had reported their tinnitus as tonal, which had also been found by other researchers [45]. However, due to the fact that subjects 11÷14 could not determine whether their tinnitus had a tonal or noise character, the subjects were examined twice using two independent tests.

Also, Table 1 contains the results of the evaluation of the characteristic features of tinnitus when using a clinical audiometer in comparison with a synthesizer. The differences are related to both acoustic parameters and the subjective evaluation of the resemblance of tinnitus to the resultant pattern and the time of the test.

In order to accurately evaluate the usefulness of the sound synthesis method discussed in this Section one should focused on the statistical evaluation of the relevance of the obtained results. The calculations were aimed at determining whether the testing of the subjects proceeds faster and whether the resulting noise pattern is subjectively more similar to the perceived tinnitus by comparing the results obtained through the use of an audiometer and synthesizer. For the calculations above the significance level of α = 0.05 was used. Calculations were made employing STATISTICA10 software. The results after statistical evaluation are shown in Table 2.

**Table 2:** Results of the statistical evaluation of tests

	**Evaluation of the resemblance of the generated noise to the participant’s noise Tinnitus in scale of [0…10]**		**Test duration [min]**	
**Participant no.**	**Audiometer**	**Synthesizer**	**Audiometer**	**Synthesizer**
1	7	9	4	2
2	6	9	6	2
3	3	8	4	2
4	2	9	5	4
5	3	3	8	6
6	6	9	10	8
7	7	9	6	2
8	9	10	6	1
9	8	8	5	2
10	8	5	3	4
11	5	5	3	2
12	7	8	5	3
13	7	8	9	4
14	1	9	2	6
Results of the Shapiro-Wilk test	**W = 0.909**	**W = 0.779**	**W = 0,945**	**W = 0,856**
	***p*** **= 0.153**	***p*** **= 0.003**	***p*** **= 0.492**	***p*** **= 0.027**
Results of the Wilcoxon test	**T = 7.0**		**T = 13.0**	
	**Z = 2.31**		**Z = 2.48**	
	***p*** **= 0.020**		***p*** **= 0.013**	
	***r*** **= 0.43**		***r*** **= 0.47**	

Key: W–Results of the Shapiro-Wilk test, T, Z–Results of Wilcoxon test, *p*–statistical significance (*p*-value), *r–*effect size.

First, the distribution of the analyzed variables was checked with the Shapiro-Wilk test. According to the calculations, both variables obtained in the audiometer test fulfill the condition of normal distribution (*p* = 0.153 and *p* = 0.492, which means that *p* > *α*), while both of the analyzed synthesizer variables do not meet the conditions of normal distribution (*p* = 0.003 and *p* = 0.027, which means that *p* < *α*). The lack of normality for the variables obtained through the use of the synthesizer didn’t allow performing a statistical evaluation of the results using the student’s *t*-test. This is why we have compared the results using an alternative method (i.e. the Wilcoxon test), which involves the sequence of pairs and is used in situations when the same variable is measured twice in varying conditions. In the discussed example, these varying conditions involve the measurements using the audiometer and the synthesizer.

Wilcoxon Signed-rank is intended for checking the significance of the differences between two dependent measurements. Having at disposal results of paired observations (audiometer and synthesizer), the Wilcoxon Signed-rank test was utilized to disclose differences between the two measurements. The test is to calculate the rank for each value, but calculate them based on the differences between the two groups. To perform the Wilcoxon Signed-rank test one should assume that all the data are paired.

Under the null hypothesis of the Wilcoxon Signed-rank test, observations that are compared come from the same population (i.e. having the same distributions). Intuitively, if there is a significant difference between two samples, the test will reject the null hypothesis. As the name of the Wilcoxon Signed-rank test suggest, it takes into account sign, differences between measurements and rank. If two scores are the same, then the pair is ignored. If two values of the difference are tied, they are given the mean of the ranks they would have had if they had been different in value. Each rank is given the sign of the difference it corresponds to. The sum of the positive and negative ranks is found. The smaller of these two sums is the test T-statistic result, which after comparing it with the critical value decides whether the null hypothesis is to be rejected.

The result of the Wilcoxon Signed-rank test is typically complemented by the evaluation of the effect size of the observed differences. The effect size coefficient (*r*) for the Wilcoxon Signed-rank test is given by the formula:

$$r=\frac{z}{\sqrt{n1+n2}}$$

where:

Z–Wilcoxon test result;

*n*_1_, *n*_2_–total number of the observations that were obtained

When assessing the effect size, the standard value of ***r*** for small size is 0.1, medium 0.3, and *r* equals 0.5 represents large sizes.

Comparing the value *p* = 0.02 obtained through the Wilcoxon test based on T-statistics with a significance level of *α* = 0.05 revealed that there is a statistically important difference in the evaluation of the similarity between the generated noise and the patient’s perceived tinnitus and the magnitude of the observed difference is medium, *r* = 0.43. Comparing the value *p* = 0.013 obtained through the Wilcoxon test based on T-statistics with significance level *α* = 0.05 revealed that there is a statistically important difference between the length of the test performed with an audiometer and a synthesizer and the magnitude of the observed difference is medium, *r* = 0.47 (see Table 2).

**Figure 2:**
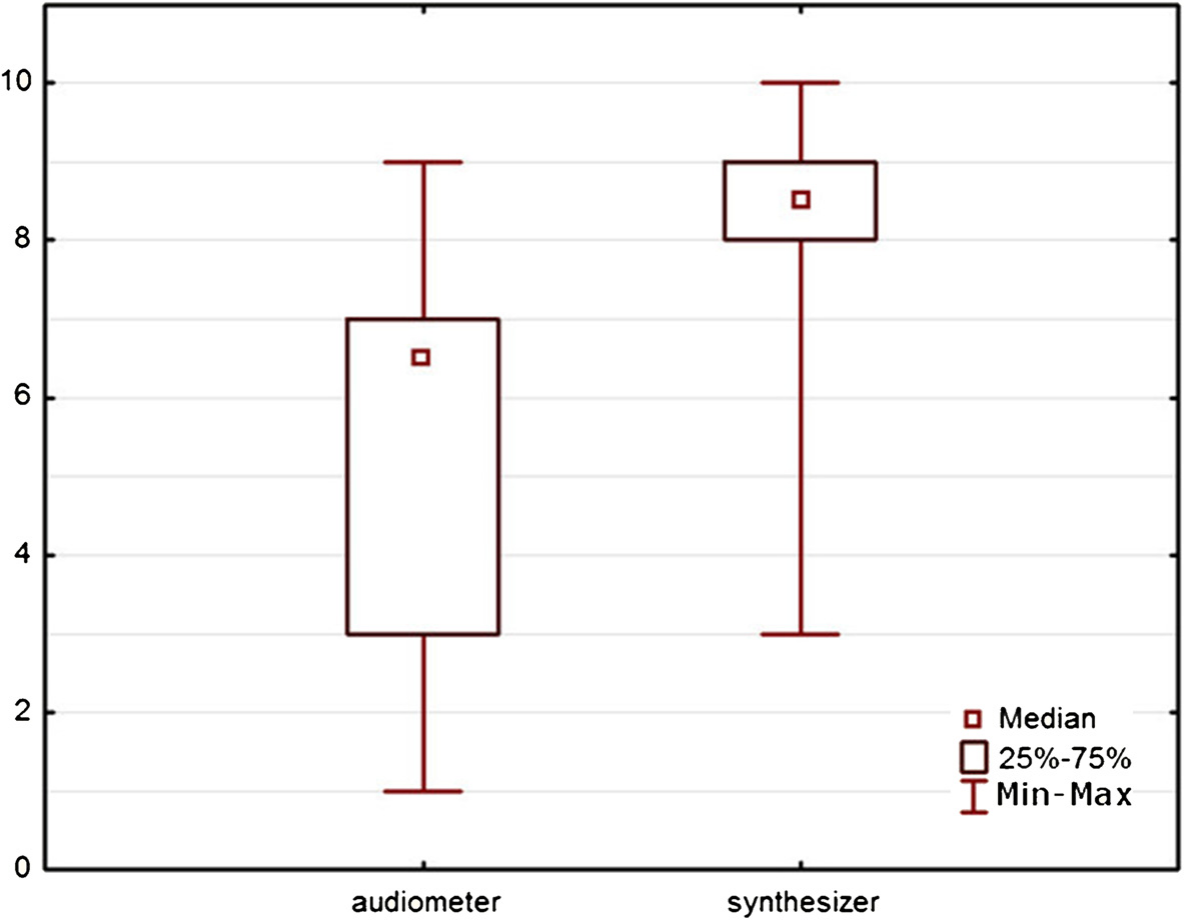
Evaluation of the resemblance of the generated noise to the noise perceived by the patient.

**Figure 3:**
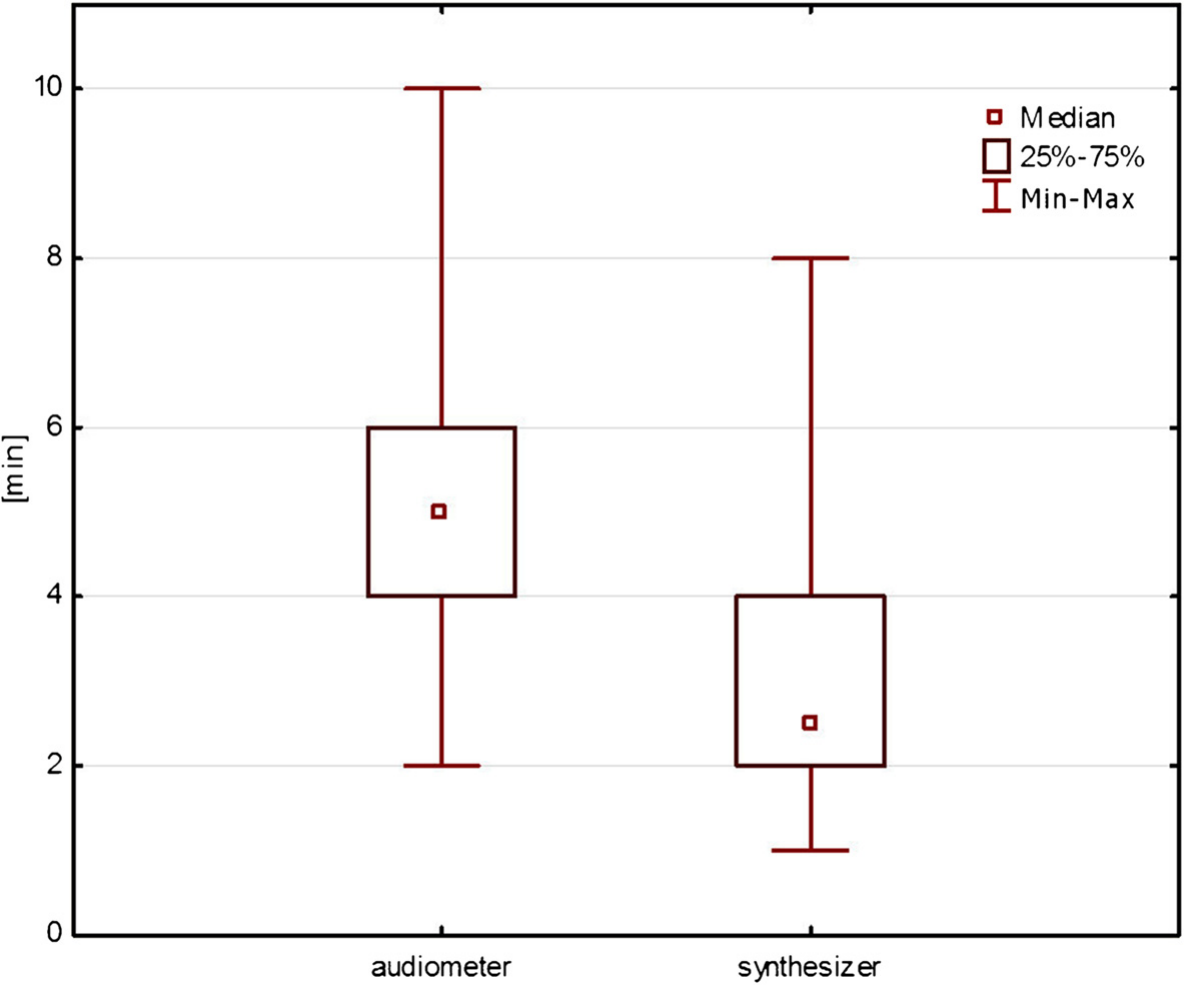
**Comparison of the length of the test using an audiometer and synthesizer.** Wilcoxon test description (http://www.uwe.ac.uk/hlss/llas/statistics-in-linguistics/chapter%2008.pdf, link from Nov. 26).

Figures 2 and 3 illustrate the distribution of the responses of the patients tested using a box plot, called also the “box and whisker plot”. The length of the box (frame) covers 50% of all observations. The so-called “whiskers” extend from the box to the highest and lowest values of the examined variable. As shown in Figure 2, the evaluation of the similarity of the noise generated using a synthesizer is greater in comparison with the noise generated by an audiometer, while exhibiting less dispersion with regards to answers.

Figure 3 illustrates the difference in the length of the test using an audiometer and a synthesizer. As can be seen, the use of synthesizer shortens the time necessary for obtaining a pattern which is the closest to the noise perceived by the patient by about a half.

## Discussion

The lower accuracy of the results in audiometer-based tests may be related directly to the limitations of the audiometer. Most diagnostic audiometers provide only a limited set of frequencies (125, 250, 500, 750, 1000, 1500, 2000, 3000, 4000, 6000, 8000), so to determine tinnitus one most often have to compromise and choose a pitch that has not been indicated by the participant. This restriction can be overcome with a clinical audiometer which provides a sweep frequency mode of operation–but that increases time needed for tinnitus determination. The second limitation is the volume level increment used in audiometers (typically 5 dB). It sets the precision with which the tinnitus loudness can be determined. Although one can change the sound level by 1 dB, it will extend the test duration.

For people who described their tinnitus as squeal, whistle, etc., the use of the synthesizer enables them to get the results more than twice as fast as with the audiometer. This is probably because people using the synthesizer look for the desired stimulus themselves and compares it to the sound they hear in their heads or ears.

When analyzing Table 1, one may notice that patient/participant no. 4, aged 79 was able to model her listening experience according to three components. In addition, she rated the obtained results by 7 points better than those obtained with the audiometer. The conclusion is that patients with tinnitus similar to hum, hiss, etc. need more time to identify their perception. However, the results obtained with the synthesizer are clearly better than those obtained using, for instance, a narrowband noise from an audiometer whose bandwidth cannot be changed due to hardware restrictions.

The case of patient/participant no. 5 shows that in some cases it is not possible to determine the acoustic parameters of tinnitus with the equipment used. Although the subject was a young, communicative and computer-literate person, he was not able to generate a sound that resembled his tinnitus to a satisfactory degree, even with multiple attempts. Eventually, he assessed the similarity of the generated sound at 3 points which is low. This may stem from the fact that the psychoacoustic evaluation of the majority of subjective tinnitus differs from the external sounds. What is more, it may be due to the fact that tinnitus results from the perception of a neural signal, which is produced in processes that are different from normal stimulation of the ear and the auditory canal by external sound. Another condition which could explain the difficulty the patient had in determining the parameters of his tinnitus is that the DPOAE test results indicated the absence of otoacoustic emissions and thus most likely the damage of hair cells in a wide frequency range (2.6 kHz-9 kHz). Perhaps the tinnitus heard by the patient has multiple components, which he cannot describe, define or distinguish. For most other participants in the test (patients no. 1, 2, 3, 6, 7, 9, 11, 12, 13 and 14), the frequency of the indicated tinnitus is correlated with the area of the absence of otoacoustic emissions in the DPOAE test, and the width of the damaged area does not exceed one octave. In accordance with the criteria of Lonsbury-Martin et al. and Dhar et al., otoacoustic emission was considered to be present if its value was higher by 3 dB than the background noise [46,47]. Lind’s [48] criterion for DPOAE presence incorporates values higher by 2 dB than the background noise. Thus in the latter case even more reliable evaluation criteria were selected. This is also confirmed by the results of the research study conducted by the Authors.

## Conclusions

The tests reveal capabilities, limitations, advantages and disadvantages of both methods for the determination of tinnitus. They show that determining tinnitus with the use of an audiometer takes half longer in most cases and it is also less accurate than with the synthesizer prepared for this purpose. This can be seen in the differences in the frequencies of the described tinnitus. The amount of time needed to determine tinnitus with the audiometer results from:

a. the need for cooperation between the participant and the person conducting the test,

b. the difficulty patients have with describing and defining their listening experience, which makes the test longer,

c. the length of the test which makes it a little tiresome

On the other hand, when using the synthesizer the patient does not have to describe verbally the perceived listening experience. Such description is usually difficult for many people–especially the elderly. In addition, there is no need for a close cooperation between the participant and the person conducting the test, which shortens the procedure. Moreover, the fact that the patients operate the program themselves makes them more involved and makes them feel more ‘responsible’ for it.

However, the more complex the description of the perceived tinnitus, the harder it is to determine the sound parameters of the patient’s perception. It also takes more time regardless of the method. The synthesizer, however, with its greater capacity for assigning the acoustic parameters of sound, represents it more precisely.

The biggest problems were to translate descriptions made by the patients on the generated sound parameters. In addition, patients often changed their minds during the test–the same sound once appeared to be identical with perceived Tinnitus and a few minutes later significant changes were indicated by the patient. This may occur due to the fact that Tinnitus is a phenomenon generally difficult to describe.

It should be emphasized that the level of patient’s cooperation during diagnosing has a huge impact on the accuracy of the diagnosis of hearing and Tinnitus in particular. The idea of the tool described in this paper was to find a way that will clearly describe the nature of the Tinnitus perceived by the patient. Taking advantages of the multimodal user-friendly interfaces and intelligent processing of data, it was possible to build the application which uses the sound synthesis to produce the sound most similar to the Tinnitus perceived by the patient.

## Abbreviations

MML: Minimum masking level; LDL: Loudness discomfort level; UCL: Uncomfortable level; MTT: Most troublesome tinnitus; TRT: Tinnitus retraining therapy; ABLB: Alternate binaural loudness balance; SL: Sensation level; 2AFC: Two-alternative forced-choice; DPOAEs: Distortion product otoacoustic emissions; RE: Right ear; LE: Left ear; Favg: Frequency average of noise; NB: Audiometric narrow band noise.

## Competing interests

The authors have declared that no competing interests exist.

## Authors’ contributions

Both authors read and approved the final manuscript. Conceived and designed the experiments: BK, TP. Performed the experiments: TP. Analyzed the data: BK, TP. Wrote the paper: BK, TP. Revised the paper: BK.

## Authors’ information

BK is professor of the Electronics, Telecommunications and Informatics Faculty and Head of the Audio Acoustics Laboratory. She is a member of the Polish Academy of Sciences and a Fellow of the Audio Engineering Society. TP is a Ph.D. student working on his thesis on tinnitus patients’ testing and rehabilitation.
